# Correlating the Integral Sensing Properties of Zeolites with Molecular Processes by Combining Broadband Impedance and DRIFT Spectroscopy—A New Approach for Bridging the Scales

**DOI:** 10.3390/s151128915

**Published:** 2015-11-13

**Authors:** Peirong Chen, Simon Schönebaum, Thomas Simons, Dieter Rauch, Markus Dietrich, Ralf Moos, Ulrich Simon

**Affiliations:** 1 Institute of Inorganic Chemistry (IAC) and Center for Automotive Catalytic Systems Aachen (ACA), RWTH Aachen University, Landoltweg 1, 52074 Aachen, Germany; E-Mails: simon.schoenebaum@ac.rwth-aachen.de (S.S.); thomas.simons@rwth-aachen.de (T.S.); 2 Department of Functional Materials, Bayreuth Engine Research Center (BERC) and Zentrum für Energietechnik (ZET), University of Bayreuth, Universitätsstraße 30, 95440 Bayreuth, Germany; E-Mails: Functional.Materials@uni-bayreuth.de (D.R.); Functional.Materials@uni-bayreuth.de (M.D.); Functional.Materials@uni-bayreuth.de (R.M.)

**Keywords:** impedance spectroscopy, microwave cavity perturbation, DRIFTS, ZSM-5 zeolite, gas sensing, ammonia, DeNO_x_-SCR, proton motion, polarization, *in situ*

## Abstract

Zeolites have been found to be promising sensor materials for a variety of gas molecules such as NH_3_, NO_x_, hydrocarbons, *etc.* The sensing effect results from the interaction of the adsorbed gas molecules with mobile cations, which are non-covalently bound to the zeolite lattice. The mobility of the cations can be accessed by electrical low-frequency (LF; mHz to MHz) and high-frequency (HF; GHz) impedance measurements. Recent developments allow *in situ* monitoring of catalytic reactions on proton-conducting zeolites used as catalysts. The combination of such *in situ* impedance measurements with diffuse reflectance infrared Fourier transform spectroscopy (DRIFTS), which was applied to monitor the selective catalytic reduction of nitrogen oxides (DeNO_x_-SCR), not only improves our understanding of the sensing properties of zeolite catalysts from integral electric signal to molecular processes, but also bridges the length scales being studied, from centimeters to nanometers. In this work, recent developments of zeolite-based, impedimetric sensors for automotive exhaust gases, in particular NH_3_, are summarized. The electrical response to NH_3_ obtained from LF impedance measurements will be compared with that from HF impedance measurements, and correlated with the infrared spectroscopic characteristics obtained from the DRIFTS studies of molecules involved in the catalytic conversion. The future perspectives, which arise from the combination of these methods, will be discussed.

## 1. Introduction

Zeolites are crystalline, microporous solids bearing tetrahedral TO_4_ (T denotes as Si, Al, Ti, *etc.*) units as primary building blocks. These corner-sharing building blocks form a three-dimensional framework with interconnected cages and channels of distinct sizes and shapes. To date, there are more than 200 framework types known according to the database of the International Zeolite Association [1]. Among these different structure types, about 17 frameworks, such as MFI, FER, MOR, LTA, BEA, FAU, or CHA are of commercial interest, e.g., as catalysts, adsorbents, sensors, or solid electrolytes [2,3,4,5].

Several important applications of zeolites are related to their Brønsted acidity, which results from the substitution of a Si T-atom with, most commonly, an Al T-atom in the primary tetrahedral TO_4_ unit. This non-equivalent (Al^3+^ ↔ Si^4+^) substitution induces an overall negative charge in the framework, which requires charge-balancing by external cations (Na^+^, NH_4_^+^, H^+^, *etc.*) in the adjacent oxygen sites within the pore space. These electrostatically bound cations are exchangeable, which leads to a series of characteristic functions, such as ion-exchange capacity, proton donating ability or ionic conductivity, being crucial for the utilization of zeolites as adsorbents, ionic conductors, sensors, or catalysts. For example, in both catalytic cracking and methanol-to-olefin (MTO) reactions, proton-form zeolites as solid acid catalysts can provide protons to react with hydrocarbons forming carbocations, which are indispensable intermediates for both reactions [5]. In proton-exchange membrane fuel cells (PEMFC), zeolites and surface-functionalized zeolites are applied as an additive of proton-conducting membrane to enhance proton conduction at high temperatures and to decrease fuel crossover [2,6]. The interaction between zeolite and specifically adsorbed guest molecules leads to a change in cation motion and consequently the electric properties of the zeolites, which can be qualitatively and quantitatively analyzed by means of impedance spectroscopy (IS), potentiometry, or microwave-based methods. Based on this principle, efficient sensors using zeolites, either as active functioning elements or as auxiliary elements in a sensor system, have been developed for the sensing of a variety of molecules including H_2_O, hydrocarbons, H_2_, CO, CO_2_, NH_3_, NO_x_ [4,7,8,9,10,11,12]. 

In the last few years, both low-frequency (100 mHz–10 MHz; LF) and high-frequency (1 GHz–10 GHz, also denoted as microwave region; HF) impedance spectroscopies have been applied to study the electric properties of zeolites [7,13,14,15,16,17,18,19,20,21,22,23,24,25,26,27]. Detailed investigations combining both experimental and theoretical methods were performed to understand the influence of framework type (e.g., FAU, MFI, CHA), Si/Al ratio (10–5000), and cation type (e.g., H^+^, Na^+^) on the proton motion in zeolites [14,16]. Further studies demonstrated that the proton conductivity of zeolites was enhanced upon interaction with NH_3_ and dependent on the NH_3_ concentration, temperature and the Si/Al ratio of the zeolites [19]. By combining low-frequency IS and temperature-programmed desorption (TPD), a mechanistic description of the NH_3_-supported proton transport processes in zeolites has been developed [21]. These fundamental understandings allowed developing efficient zeolite-based impedimetric NH_3_ sensors for different purposes [7,18,28]. 

One of the emerging applications is the *in situ* monitoring of zeolite catalysts for selective catalytic reduction of nitrogen oxides (DeNO_x_-SCR). When NH_3_ serves as the reductant, the SCR reactions are described as follows:

4 NH_3_ + 4 NO + O_2_→4 N_2_ + 6 H_2_O (standard SCR) (1)

2 NH_3_ + NO + NO_2_→2 N_2_ + 3 H_2_O (fast SCR) (2)

8 NH_3_ + 6 NO_2_→7 N_2_ + 12 H_2_O (NO_2_ SCR) (3)

It is accepted that during SCR, NH_3_ is stored in zeolite catalysts and then activated by Brønsted acid sites forming reactive NH_4_^+^ species for NO_x_ reduction [29]. The presence of NH_3_ species changes the proton conductivity of the zeolite catalysts [19]. By monitoring the proton conductivity of zeolite catalysts upon loading, desorption or consumption (by SCR reaction) of NH_3_ using *in situ* low-frequency IS, a correlation between the NH_3_ storage and the change in proton conductivity for H-ZSM-5 and Fe-ZSM-5 SCR catalysts was established [25,26]. *In operando* investigations using microwave-based methods revealed that the resonance frequency of a commercial zeolite catalyst is quantitatively related to the amount of the stored NH_3_ [27]. These preliminary studies demonstrate the great potential to utilize zeolite catalysts as sensors for the monitoring of SCR reactions under technically relevant SCR conditions [24,27]. Based on these achievements, a special laboratory setup for dielectric characterization of catalyst materials to separate polarization and dielectric losses under reaction conditions was developed [20]. With this new setup, new information about the response of the complex dielectric permittivity to ammonia in H-ZSM-5 zeolites with varying Si/Al ratios [30], and of H-form and Cu-exchanged ZSM-5 zeolites in a comparative study [31] has been obtained.

**Figure 1:**
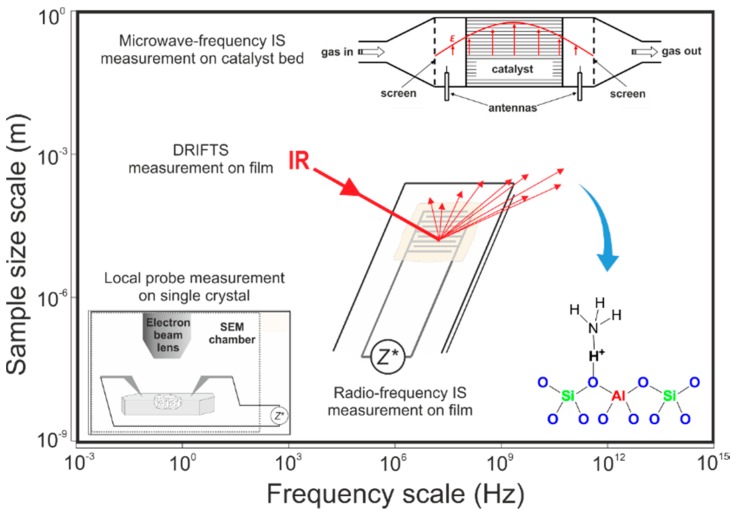
Schematic representation of the spectroscopic techniques at different working frequency ranges and of the probe at different dimensions. It thereby illustrates the different scales of size and frequency, which are assessed by the different experimental techniques described in this work.

While IS over a broad frequency range from mHz to GHz is suited to analyze the integral conductivity of zeolites, extending the electromagnetic frequency spectrum for spectroscopic analyses to THz, *i.e*., towards infrared spectroscopy, enables probing molecular events on the zeolite surface (Figure 1). To further understand the NH_3_-supported proton transport on a molecular level, a setup combining the IS with diffuse reflection infrared Fourier transformation spectroscopy (IS-DRIFTS) was developed, which allows us to simultaneously monitor both the real-time electric state of the zeolite and the vibration modes of the molecules involved in the DeNO_x_-SCR reactions. The combination of these spectroscopic techniques in broadband frequency thus not only expands our understanding of the sensing properties of zeolite catalysts from integral electric signal to molecular processes but also bridges the length scales under study from centimeters to nanometers.

In this review, we will briefly introduce the physical background, the instrumentation and the data analysis for both the low-frequency (LF) and high-frequency (HF) IS when applied to zeolites (Section 2). In Section 3, we will introduce the recent developments of zeolite-based sensors in gas sensing and reaction monitoring, and compare the electrical impedance properties at both frequency ranges. The low frequency electrical response and infrared spectroscopic characteristics obtained from the molecules involved in the catalytic conversion will be correlated. In Section 4, the future perspectives which arise from the combination of these methods will be discussed.

## 2. Instrument Development

### 2.1. Low-Frequency Impedance Spectroscopy

The charge-conducting behavior in zeolites was reported as early as 1930s, and was initially attributed to the presence of mobile cations [32]. In the 1960s and 1970s, IS was applied to investigate the electrical properties of zeolites (dehydrated Faujasite) [33,34,35,36]. 

IS, based on perturbation theory, allows evaluating the mobility of cations in zeolites. In IS, an alternating voltage *U* with angular frequency ω and amplitude *U*_0_ is applied to a zeolite in thermodynamic equilibrium, thus forming an electric field over the zeolite. This electric perturbation induces a response of the system, *i.e*., a movement of the mobile cations either via translation motion or a local displacement, which can be macroscopically measured as a current *I*. The complex impedance *Z*(ω) is defined as the ratio between the applied complex voltage *U* and the resulting current *I*, that is,

*Z*(ω)* = U*/*I* (4)

*Z*(ω) can be described by a real part *Z*´ and an imaginary part *Z*´´. Both depend on the angular frequency ω.

*Z*(*ω*) = *Z´*(ω) *+ jZ´´*(ω) (5)

Hence, a decrease in the mobility of cations induces a decreasing current (at constant applied voltage *U*) which results in a rising impedance *Z* [37].

By means of IS, it was possible to develop a mechanistic picture of the ionic conductivity, confirming the hypothesis of Rabinowitsch *et al*., *i.e*., the ionic conductivity of zeolite Y results from the movement of exchangeable cations by overcoming potential barriers of different height. Additionally, a local motion of cations within α-supercages occurs, which is superposed by the ionic conductivity of the material [35]. Nevertheless, the exact influences on and calculations of the potential barrier height were not fully achieved till the 1990s.

**Figure 2:**
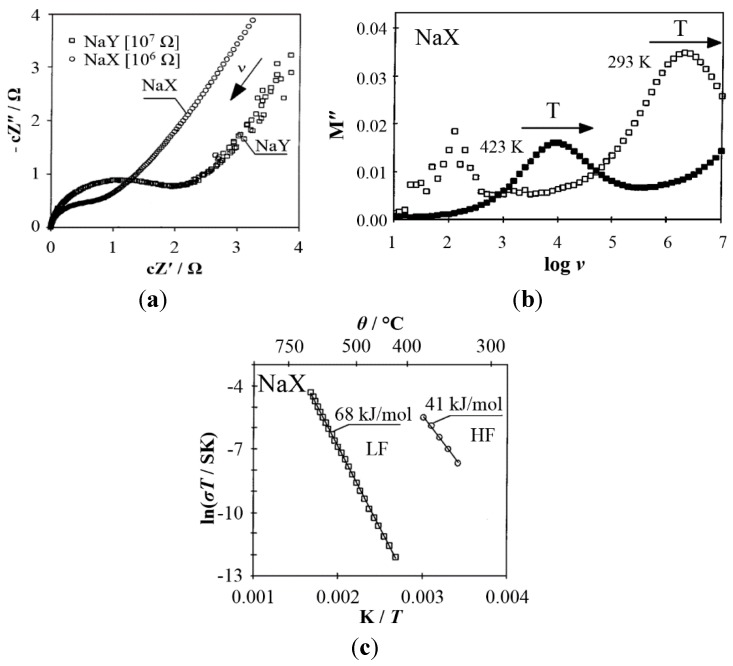
(**a**) Argand diagram (plot of the scaled imaginary part of the impedance *cZ"* *versus* the real part of the impedance *cZ′* in the complex plane) of dehydrated NaX and NaY at 573 K; (**b**) Modulus spectra of the imaginary part *M"* *vs.* log *ν* of NaX (at 293 and 423 K); (**c**) Arrhenius-like plots of NaX and the activation energies for the respective processes. Adapted from [39] with permission from Springer.

In 1998, a new evaluation method for impedance data was applied, which allowed a more detailed understanding of cation mobility in zeolites [15,38]. Commonly, the impedance data are plotted as *Z´* against *Z*″ of *Z*(*ω*) in the so-called Argand plot (also known as Nyquist plot), of which an example is shown in Figure 2a. Low-frequency phenomena such as the sample/electrode interface polarization are easily identified because they are represented by the dominating low-frequency tail, which increases with decreasing frequency down to the quasi-DC limit. In contrast, the so-called modulus plot, which shows the imaginary part of the modulus against the frequency, is more suited to illustrating the high-frequency processes (Figure 2b). The modulus *M*″(ω) is defined as

*M″*(ω) =ωC_0_*Z´*(ω) (6)

where *C_0_* is the capacity of the empty capacitor, *i.e*., the geometric capacitance.

Thus, more weight is given to the high-frequency part in modulus plots, so that relaxation processes at high frequencies are more visible [39,40]. The modulus plot shows a maximum at the resonance frequency ***ν*_res_** of the system, which is the inverse of the time that a perturbed system needs to reach thermodynamic equilibrium again, or shortly the relaxation time ***τ*** = *R*·*C*, where *R* is the resistive part and *C* is the capacitive part of the respective relaxation process. ***τ*** undergoes a high frequency shift with increasing temperature when the underlying conductivity process, which causes the resistive part, is thermally activated, while the capacitive part remains often almost constant, since the capacitive contribution to the electrical response is usually less temperature-dependent than the conductivity. From the thermal shift, the activation energy *E*_A_ of the conducting process can be derived from an Arrhenius-like plot (Figure 2c), as

ln[*Y′_ν_*_res_(*T*)] ~ ln[*σ*(*T*)] = ln(σ_0_/*T*) + *E*_A_/(*k*_B_*T*) (7)

where *Y′_ν_res__* (*T*) is the real part of the admittance at the temperature-dependent resonance frequency, *σ* is the specific conductivity of the zeolite, and *k*_B_ is the Boltzmann constant.

**Figure 3:**
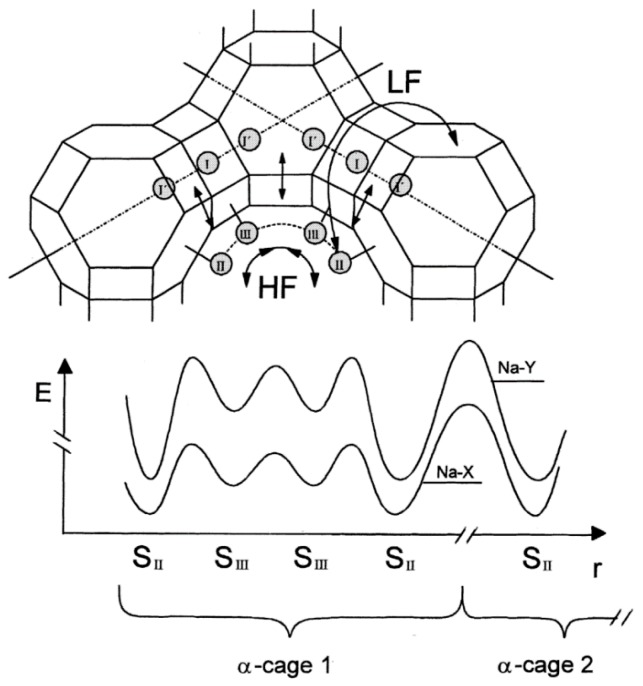
Illustration of the conductivity processes in Faujasite zeolite and the corresponding potential profile indicating the different activation energies of the high-frequency and low-frequency processes [39]. Reprinted with permission from Springer.

This method of impedance data analysis helps to visualize and distinguish two distinct relaxation processes, *i.e*., the local dipolar relaxation and the long-range charge transport, within one spectral representation (Figure 3) [39]. By analyzing different samples, it was found that the *E*_A_ for each process depends on the zeolite structure and thus includes features of cation-cation interactions. It thereby demonstrated that from the macroscopic response and by including structural details, elementary proton motion processes on the microscopic scale could be deduced.

Based on these results, Franke *et al.* studied the proton conducting processes in H-ZSM-5 by means of IS, and calculated the activation barriers and jump rates for translational proton motion using a combined quantum mechanics–interatomic potential function approach (QMPot) [41,42]. These experimental and theoretical investigations disclosed that a decrease in the Si/Al ratio leads to a decrease in the distance-dependent activation energy for proton motion, suggesting the same mechanism of proton transport in zeolites, *i.e*., proton hopping. Although the values of activation energies for proton motion in zeolite structures deviate significantly with Al–Al distances, impedance measurements were found to be able to detect the complete translational proton motion between neighboring Brønsted sites [43,44].

**Figure 4:**
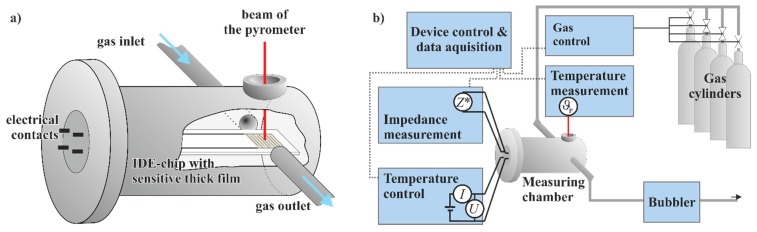
(**a**) Measuring chamber; and (**b**) the overall measuring setup for impedance measurements on IDE structures with gas-sensitive thick film layers.

In following studies, Franke *et al.* [18,42] and Rodriguez-Gonzales *et al.* [21,45,46] investigated the influence of the host-guest interactions between zeolites and gases, such as NH_3_ and water vapor, on proton mobility, and discussed the working principle of zeolite-based NH_3_ sensors (see Section 3). For these measurements, a heatable sample holder with an interdigital electrode (IDE) structure was applied, similar to that shown in [47]. Utilization of IDE structures enhances the contact between the investigated gases and the zeolites, thereby increasing the signal response of the zeolites and allowing the development of a new IS measurement setup (see Figure 4). By this means, it is possible to heat the zeolite thick film to temperatures above 500 °C under flowing gas conditions in a measuring chamber. For temperature control, an external power supply is used, which also measures the resistance of the heating element. For each sample, the temperature is calibrated using a pyrometer. The gas composition is controlled by mass flow controllers (MFCs) dosing different gases such as NO, O_2_, NH_3_ and N_2_ (carrier gas), respectively. The electrical impedance of the sample is measured with an impedance analyzer range up to 10^14^ Ω (±1%). The voltage is set to 0.1 V (rms) for all measurements to stay in the linear regime.

### 2.2. High-Frequency Impedance Spectroscopy

**Figure 5:**
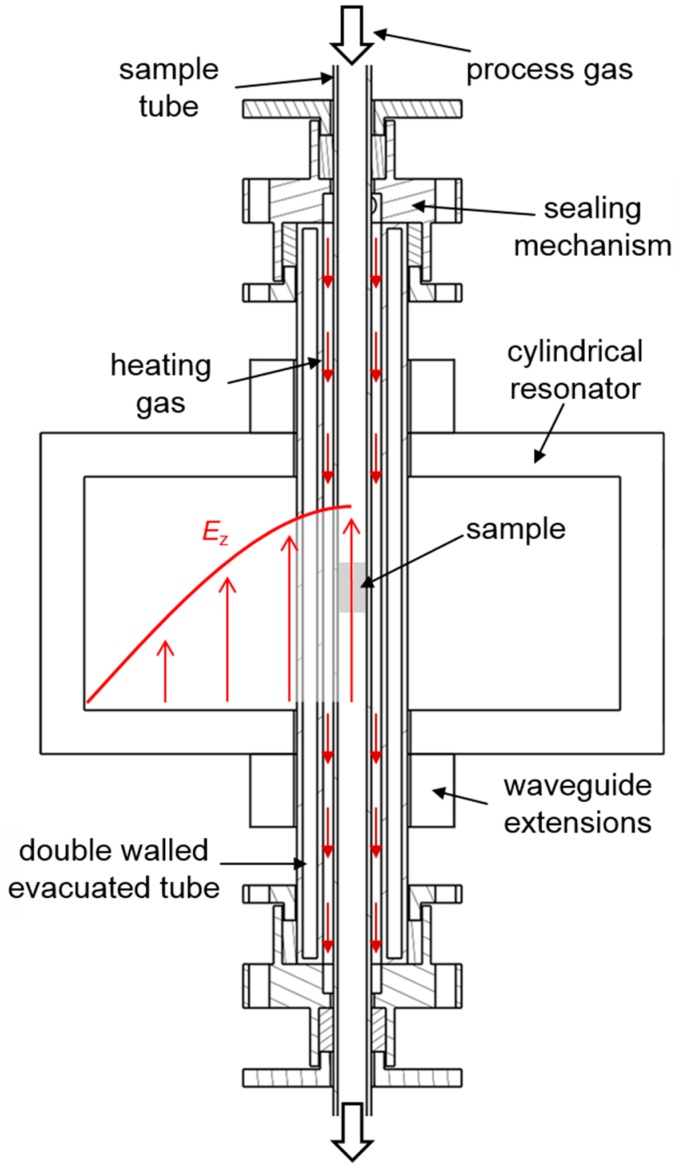
Scheme of the test setup including the sample tube, the glass tube system with sealing mechanism, the resonator with waveguide extensions, loop antennas, and two thermocouples. Reprinted from [20].

For the *in situ* determination of dielectric properties (including polarization, dielectric losses and conductivity effects) of zeolites and other catalytically active materials in the high-frequency (GHz) range, the microwave cavity perturbation method is the tool of choice, with the benefit of being contactless and noninvasive [48,49,50,51]. In this technique, a hollow metal cavity resonator is used. By coupling electromagnetic waves into the resonator, standing waves (resonances) appear at specific frequencies. Inserting a sample inside the resonator changes the dielectric properties of the high electric field, which can be determined by analyzing the resulting decrease in resonance frequency and increase of the 3 dB bandwidth (*i.e*., decrease of the quality factor *Q*) [52]. Especially for this purpose, a measurement setup as fully described in [20] was developed (schematically displayed in Figure 5), using a cylindrically shaped, inductively coupled resonator. The resonator is designed to analyze the TM_010_ mode (around 1.2 GHz, electrical field shown in red in Figure 5) with its uniform electrical field maximum along the cavity’s axis. In this field maximum, the powder samples are placed on a porous frit in a multi-walled glass quartz system, which guides two separated gas streams: the outer stream for heating the sample to reaction temperatures and the inner stream for the process gas, *i.e*., synthetic exhaust gas controlled by mass flow controllers. The downstream gas concentrations are monitored by a Fourier Transform Infrared Spectrometer (FTIR) analyzer. For a constant cavity temperature, the cavity is permanently water-cooled.

The complex dielectric permittivity of the sample placed in high electric field can be determined by comparing the resonance system without and with sample. Therefore, reference spectra for each set temperature are required. Both parts of the complex permittivity *ε* = *ε*′ – *jε*" can be attributed to specific resonance characteristics: polarization effects, quantified by the real part *ε*′ (or, more properly, by *ε*′ − 1), are mirrored in the change of the resonance frequency from *f*_0_ (without sample) to *f*_s_ (with sample); the effects of dielectric loss, quantified by the imaginary part *ε*", are related to the change of the quality factor from *Q*_0_ (without sample) to *Q*_s_ (with sample). The complete relationship between the permittivity and these two resonance characteristics *f* and *Q* is shown in the Equations (8) and (9). Additionally, the volume of the sample *V*_s_ (here determined by a Helium gas pycnometer) and the mode of the resonator *V*_eff_ (which is the effective volume occupied by the electric field energy and depends on the particular cavity mode; for the TM_010_ mode *V*_eff_ is 26.9% of the cavity volume) are required [53].

(8) $$\frac{f_{0}-f_{s}}{f_{0}}\approx \left(\varepsilon \prime -1\right)\frac{V_{s}}{2V_{eff}}$$

(9) $$\frac{1}{Q_{s}}-\frac{1}{Q_{0}}=\Delta \left(\frac{1}{Q}\right)\approx \varepsilon \prime \prime \frac{V_{s}}{V_{eff}}$$

**Figure 6:**
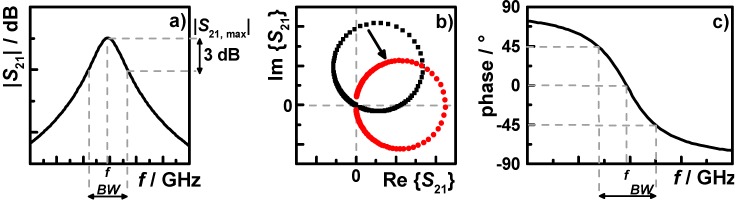
(**a**) A resonance peak in detail, showing the resonant frequency *f*, 3 dB bandwidth *BW*, and maximum peak height |*S*_21, max_| determined by a scalar data analysis approach; (**b**) the corresponding resonance circle (black) and the canonical transformed circle (red); and (**c**) the frequency-phase relation of the transformed resonance circle for the complex analysis approach for *f* and *BW*.

For the high frequency measurements, the resonance characteristics were determined by recording the transmission scattering parameter *S*_21_ with vector network analyzers (VNA). A schematic resonance peak of *S*_21_ is shown in Figure 6a with the considered parameters for microwave analysis, *i.e*., the resonance frequency *f*, the 3 dB (or “half-power”) bandwidth *BW*, and the peak height |*S*_21,max_|. 

Equation (9) requires the unloaded quality factor *Q*, *i.e*., without the effects of cavity loading. Therefore, the cavity is designed (and measured) to have symmetric coupling, by equal inductive coupling strength at each of its two ports. Then, the coupling unloading process can be calculated using Equation (10) [53].

(10) $$Q=\frac{f}{BW}\left(1-10^{-|S_{21,\max}|/20}\right)$$

To achieve a significantly higher measurement accuracy than scalar Lorentzian-type curve fitting, a complex analysis approach for microwave parameter determination can be used. Figure 6b shows the corresponding resonance circle (black) of the peak in Figure 6a. In the analysis, the circle is transformed to the ideal position (red), *i.e*., passing through the origin and centering on the real axis. The frequency-phase relation of the transformed circle (displayed in Figure 6c) is used to determine the resonance frequency and the 3 dB bandwidth using the characteristic phase values at −45°, 0°, and +45°. In addition, the peak height is determined by the diameter of the resonance circle [54,55].

**Figure 7:**
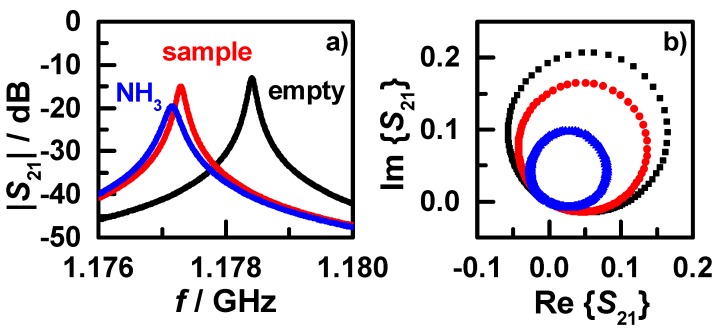
(**a**) Example resonance peaks at 200 °C of the empty sample tube (black), with inserted H-ZSM-5 zeolite sample (red), and the sample loaded with ammonia (blue); (**b**) the corresponding resonance circles.

The effects of sample insertion and NH_3_ storage on a zeolite sample on the transmission signal are displayed in Figure 7, as resonance peak (a) and resonance circle (b), respectively. The peak of the empty cavity (black) shifts to a lower frequency and decreases slightly in peak height as the sample is inserted (red). The NH_3_-saturated sample shows an additional frequency shift and a stronger decrease in peak height. Correspondingly, the resonance circle decreases in diameter and rotational angle. The latter is too small to be visible in Figure 7b, but can be determined by the explained phase analysis procedure.

Both the real and the imaginary parts of the complex permittivity of ZSM-5 zeolites change coincidentally with the loading of NH_3_ and showed different dielectric responses to the Si/Al ratio [30] and ion-exchange [31].

**Figure 8:**
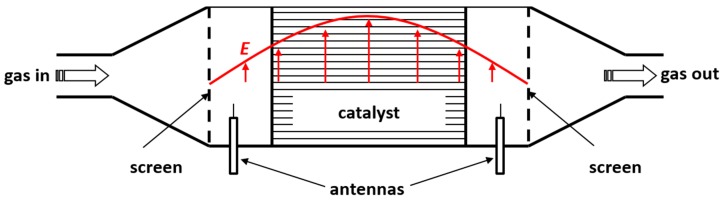
Schematic illustration of the resonator cavity for monitoring of exhaust gas after treatment devices, with antennas and screens up- and downstream of the catalyst or filter. The electrical field strength of the TE_111_ mode is illustrated in red.

Similar investigations were already performed to directly measure the state of entire exhaust gas after-treatment devices with a diameter of serial-type catalysts, *i.e*., in the 10 to 20 cm range. Here, the metal housing (canning) of automotive catalysts [23,56] and filters [57,58,59] was used as a resonator cavity (schematically displayed in Figure 8). By flanging metal screens up- and downstream of the catalyst canning, the volume of the resonator was defined. Due to the orientation of the applied coaxial capacitive probe antennas, transverse electric (TE) modes, mostly the lowest one (the TE_111_ mode), were used in these systems. The electrical field distribution of TE_111_ is displayed in red in Figure 8. Good correlations have been obtained between the observed resonance frequency and the catalyst state; for example, the oxidation state of three-way catalytic converter [60,61,62,63], NO_x_ storage in lean NO_x_ traps [64], soot or ash loading of particulate filters [57,59,63,65] and ammonia storage in SCR catalysts [24,27].

In this special application-relevant case, when the sample occupies most of the cavity volume, the electrical field strength varies over the sample length, and the material characterization Equations (8) and (9) are not valid. Nevertheless, the basic response of the resonance and the quality factor to the changes in polarization and losses of the cavity’s filling are still the same as for the ideal cavity. Therefore, simplified Equations (11) and (12) with dimensionless constants *A* and *B* can be used, since the desired information is the catalyst state but not the exact dielectric properties.

(11) $$\frac{\Delta f}{f}\approx A\left(\varepsilon \prime -1\right)$$

(12) $$\Delta \left(\frac{1}{Q}\right)\approx B\varepsilon \prime \prime$$

### 2.3. DRIFT Spectroscopy in Combination with Low-Frequency Impedance Spectroscopy

DRIFTS is a spectroscopic technique for the *in situ* study of catalytic reactions on solid materials [66]. In DRIFTS measurement, the infrared light inclining on a sample is reflected in a diffuse manner by the rough surface of the measured sample, and collected by an ellipsoid or paraboloid mirror. 

**Figure 9:**
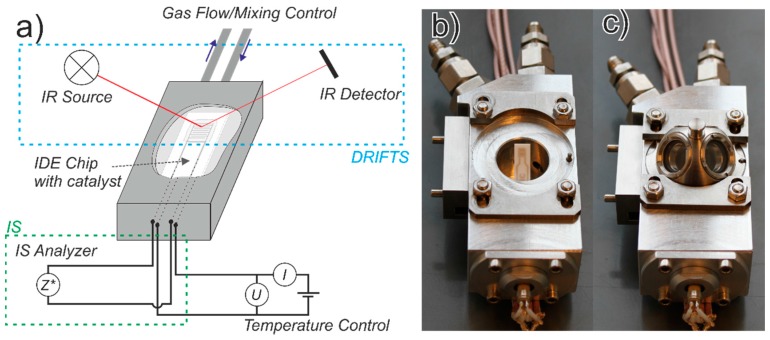
Simultaneous IS and DRIFTS measurements on the same catalyst film. (**a**) Scheme of the measurement configuration; (**b**) Photograph of open measuring chamber; (**c**) Photograph of measuring chamber with dome. Adapted from [67] with permission from Elsevier.

In order to simultaneously monitor both the proton conductivity of zeolites and the vibration modes of the molecules on zeolites, a setup combining IS and DRIFTS was designed and constructed (see Figure 9) [67]. A high-temperature reaction chamber was modified to allow the introduction of sensor chips equipped with IDE structures (125 µm spacing), on which the zeolites have been previously deposited as 50 µm thick films, and an integrated backside heater. Detailed description of the electrode design can be found elsewhere [68]. A specially designed holder with electrical contacts was employed to keep the sensor chip inside the reaction chamber in a way that the zeolite film is in the focal point of the infrared beam of the DRIFTS mirror design. Simultaneous IS and DRIFTS measurements were carried out using the same catalyst film. Gas feed into the chamber was controlled by a gas control and mixing system. The film temperature was controlled by the integrated backside heater of the sensor chip using external power source, and calibrated using a spectral pyrometer.

In order to increase the time resolution, impedance measurements at selected frequencies (close to the relaxation frequencies of the measured sample) instead of a broad frequency range were performed. The absolute value of complex admittance *|Y|* (*Y* is the reciprocal of the complex impedance *Z*, *i.e*., *Y* = *1/Z*, see above) was used for the evaluation.

The DRIFTS measurements were performed with a FTIR spectrometer in combination with a Praying Mantis mirror system for diffuse reflection spectroscopy. The spectra were recorded in the range from 4000 to 650 cm^−1^ with a resolution of 2 cm^−1^. The reflectance is given in Kubelka-Munk units (KM), which linearly correlate the band intensity and the concentration of the respective species adsorbed on the catalyst. All presented spectra are difference spectra, *i.e*., a spectrum of the pure sample under N_2_ at the respective measurement temperature was collected and was subtracted from all further spectra recorded under different gas atmospheres at the same temperature. Therefore, only the vibration modes of the adsorbed species and no lattice vibrations are visible in the difference spectra. 

In IS-DRIFTS study of zeolites as SCR catalyst, the samples were pretreated prior to any measurement at high temperatures (400–500 °C) in O_2_/N_2_ flow for a certain time to remove possible hydrocarbon contaminates on the zeolite surface. At the desired measurement temperature, the zeolite material was firstly exposed to flowing N_2_ as reference, then to flowing NH_3_ for adsorption. Afterwards, pure N_2_ or NO/O_2_/N_2_ mixture was fed to the chamber for the thermal desorption or catalytic conversion of adsorbed NH_3_ on zeolite. IS and DRIFTS signals were collected simultaneously to correlate the electrical response with surface processes. To visualize the correlation, the recorded IS and DRIFTS signals were normalized by setting the values at the initial NH_3_-free state to 0 and the values at full NH_3_ uptake to 1. Those normalized values are defined as *I_IS_* and *I_DRIFTS_*, respectively.

## 3. Development of Zeolite-Based, Impedimetric Sensors for Automotive Exhaust Gases

Impedance measurements on zeolite H-Beta have revealed that NH_3_ influences the effective proton mobility, which can be directly measured by conductivity increase in the presence of NH_3_ [15]. In case of Na^+^-form zeolite Beta, it was found that the conductivity is not affected in the presence of the guest molecule. Therefore, it was concluded that NH_3_ predominantly influences the mobility of the Brønsted acidic protons.

**Figure 10:**
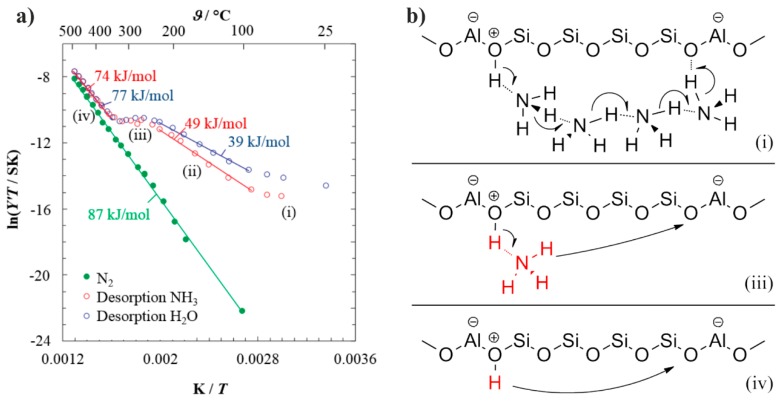
(**a**) Arrhenius-plot recorded in the presence or absence of solvate molecules. The green data points denote measurements of an initially dried sample in N_2_ environment. The red and blue data points, indicate measurements in N_2_ over samples loaded with NH_3_ and H_2_O, respectively. The straight lines indicate the ranges, from which the respective activation energies (*i.e*., the numbers along the curves) have been derived. (i)–(iv) denote different characteristic temperature ranges, which are characterized by different charge carrier transport mechanisms; (**b**) Schematic illustration of the different charge transport mechanisms occurring in the respective temperature ranges which are denoted according to the Arrhenius-plot. Adapted from [19] with permission from John Wiley and Sons.

Franke *et al.* [19,42] studied the proton mobility in H-ZSM-5. By heating zeolites fully loaded with water vapor and ammonia, they found that the activation energy of proton motion in zeolites is temperature-dependent in a characteristic manner (Figure 10). It was proposed that at low temperatures, *i.e*., at high loadings of ammonia or water, the inner surface of zeolites is covered by a condensed phase of weakly bound solvate molecules (denoted as solvent molecule chains) between adjacent Brønsted sites, which allows a Grothuss-like charge transport. Due to the abundance of adsorbed solvate molecules forming solvent complexes with the acidic protons at the Brønsted sites, proton transfer occurs with relatively low activation energy, which is not further specified due to the limited measuring points (see (i) in Figure 10). With increasing desorption of the weakly bound molecules, the above mentioned chains of solvate molecules disintegrate leading to the formation of “gaps”. Thus, a higher activation barrier has to be overcome for protons being transferred from one chain to another via such “gaps” (see (ii) in Figure 10). When the weakly bound molecules are completely desorbed, proton transport can only occur via a vehicle mechanism, with the NH_4_^+^ or H_3_O^+^ serving as vehicles for the protons and moving from one Brønsted site to the neighboring one. As compared to proton motion, the moving of such vehicle species has a significantly lower activation energy [18]. At such relatively higher temperatures, the thermally activated proton motion increases considerably the proton conductivity of zeolite as well. As a whole, an almost constant conductance was observed over this temperature range, as seen in (iii) in Figure 10. Above 340 °C, solvate molecules are desorbed and the conductance as well as the activation energy approach the values of the solvate-free zeolite, as shown in (iv) in Figure 10 [18]. Thereby, the temperature-dependent IS measurements reflect the energy landscape for the motion of proton through the polyanionic lattice structure, and how it is affected by the uptake of guest molecules, *i.e*., H_2_O and NH_3_. 

Even though water and ammonia support the proton conduction in zeolites in a similar manner, the different desorption temperatures, *i.e*., 420 °C for H_2_O and 460 °C for NH_3_, indicate a weaker interaction of H_2_O with the zeolite lattice as compared to NH_3_ [19]. For a selective impedimetric thick-film sensor for NH_3_, a better performance can be achieved by operating at temperatures above the desorption temperature of H_2_O. Despite its similar characteristic interaction with the mobile protons, H_2_O displays a weak influence on the sensing of NH_3_. Specifically, a change of 1 vol.% H_2_O vapor had the same effect on the sensor signal as 6 ppm NH_3_ [47]. 

**Figure 11:**
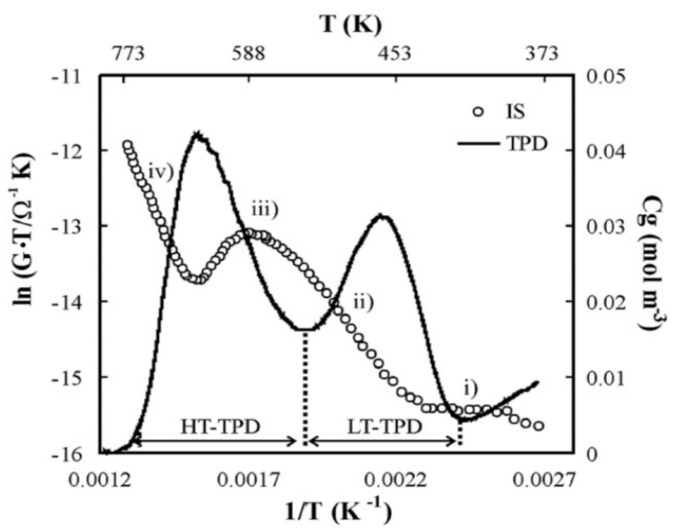
NH_3_-TPD profile and logarithm of the proton conductivity of H-ZSM-5 (Si/Al = 80) *versus* the reciprocal temperature. The plots show the thermal desorption of NH_3_ adsorbed on different sites at low and high temperatures and the corresponding change in proton conductivity. (i)–(iv) denote different characteristic temperature ranges as shown in Figure 10 [21]. Reprinted with permission from Elsevier.

Based on a fundamental understanding of host-guest interactions, Franke developed a zeolite-based, impedimetric humidity sensor [42], which was further refined by Neumeier *et al.* to detect water in the ppm range in hydrogen atmosphere at temperatures up to 600 °C [69]. Rodriguez-Gonzales *et al.* compared the characteristic features of the temperature-programmed desorption of ammonia (NH_3_-TPD) [46] and impedance measurements of different zeolite samples [28]. They showed that the two peaks observed in NH_3_-TPD correspond to characteristic changes in the charge transport mechanism (Figure 11). These results confirmed the mechanism proposed by Franke *et al*., who stated that the temperature-dependent transitions from the Grotthuss-like charge transport to the vehicle mechanism and finally to proton hopping are responsible for the conductance properties observed by IS. Further development of this sensing concept allowed the introduction of the so-called IS-TPD, which applies a highly sensitive zeolite-based NH_3_ sensor instead of a gas chromatograph as used in the conventional NH_3_-TPD. This IS-TPD setup allows a substantial reduction in the sample mass and helps minimize temperature gradients in NH_3_-TPD measurements. In automotive sensing applications, to avoid ammonia being oxidized by NO according to the SCR reaction in Equation (1), H-ZSM-5 or other catalytically inactive zeolites with a high Si/Al ratio are preferred. Such zeolite materials are relatively less conductive due to the larger distances between the Brønsted sites, and thus a high measuring frequency has to be applied to obtain a precisely measurable current [47].

The promising electrical response towards NH_3_ prompted the exploration of zeolites as a sensor to monitor zeolite-catalyzed reactions involving NH_3_, in particular DeNO_x_-SCR, where NH_3_ is the most widely used reductant source [25]. In DeNO_x_-SCR, urea solutions as NH_3_ precursor are injected into the SCR unit from a storage tank by a delivery system, and is hydrolyzed to release NH_3_ at elevated exhaust temperatures. Therefore, the injection of NH_3_ has to be adjusted in order to achieve the highest possible NO_x_ conversion and to avoid ammonia slip [70]. Thus, to further improve the DeNOx-SCR efficiency, it is of great importance to understand NH_3_ storage in zeolite catalysts under technically relevant conditions.

**Figure 12:**
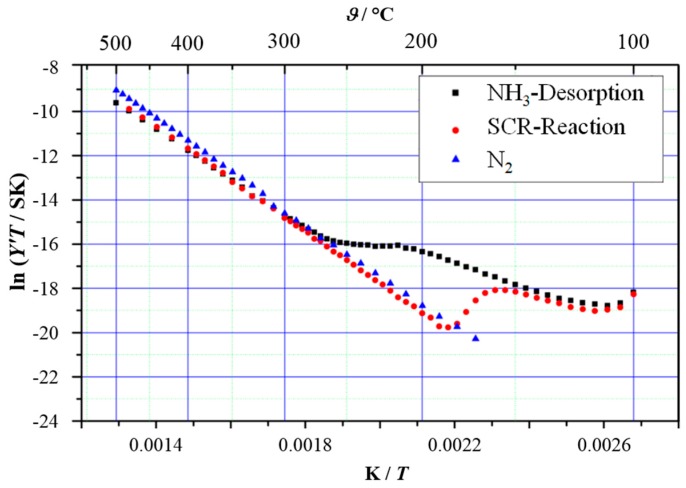
Arrhenius-like representation of the conductance as obtained by IS measurements over H-ZSM-5 zeolite (Zeolyst: Conshohocken, PA, USA; Si/Al = 80) under different gas conditions (stars: thermal desorption, *i.e*., NH_3_-solvated zeolite in N_2_; circles: SCR, *i.e*., NH_3_-solvated zeolite in NO/O_2_ mixture; triangles: reference, *i.e.* non-solvated zeolite in N_2_.). Adapted from [25] with permission from Cambridge University Press.

As described above, by applying thermal desorption conditions over NH_3_-saturated zeolites, the proton conductivity of zeolites is highly dependent on the amount of stored NH_3_ [18,21]. Performing the same IS measurement in NO/O_2_ mixture led to characteristic impedance features different from those for thermal desorption [25]. Figure 12 compares the Arrhenius-like representation of the conductance obtained by IS measurements under different conditions. For reference measurement over non-solvated H-ZSM-5 in N_2_, a linear curve was observed indicating thermally activated proton hopping. Similar measurements over NH_3_-solvated H-ZSM-5 showed increased conductivity at temperatures below 260 °C due to NH_3_-supported proton motion, in accordance to the previous studies [18,21]. Above 260 °C, similar conductivity to that of the non-solvated sample was observed due to the complete desorption of stored NH_3_. Exposing the NH_3_-loaded H-ZSM-5 to NO/O_2_ mixture instead of N_2_ did not significantly alter the conductivity at temperatures lower than 150 °C. However, further increasing the temperature led to a decay of the conductivity reaching similar *ln(Y′T)* values as those of non-solvated H-ZSM-5 at 190 °C and above. The different proton conductivities lead to a distinct “SCR window” in the Arrhenius representations. IS measurements using NH_3_-loaded H-ZSM-5 in the presence of NO or O_2_ showed similar impedance behaviors as those in N_2_, revealing that the decrease of conductivity to a non-solvated state below 260 °C results from NH_3_ conversion by NO/O_2_. Further studies showed that the SCR reaction on metal-promoted zeolite catalysts (Fe-ZSM-5, Cu-ZSM-5, Cu-SAPO, *etc.*) can be monitored *in situ* as well by means of IS, demonstrating the power of IS in the monitoring of both catalyst state and the SCR reaction.

**Figure 13:**
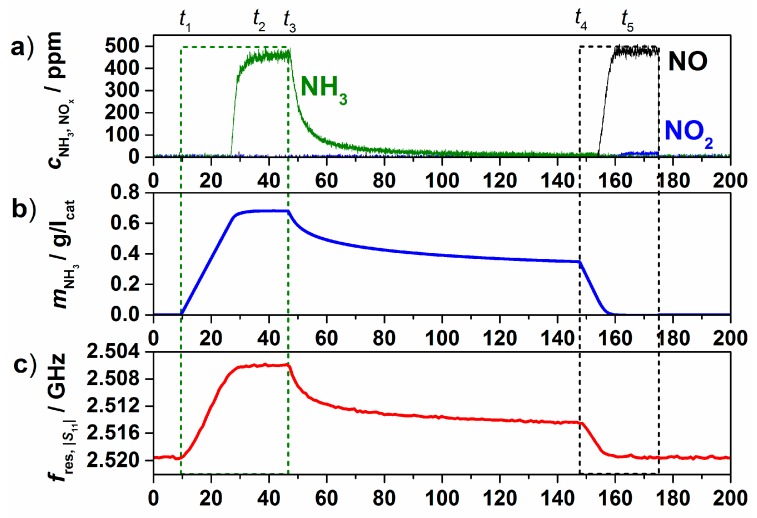
Cu-SSZ-13 honeycomb catalyst: NH_3_ storage measurement at 250 °C; (**a**) inlet (dashed lines) and outlet (solid lines) gas concentrations of NH_3_ (green), NO (black) and NO_2_ (blue); (**b**) calculated amount of NH_3_ stored on the catalyst; and (**c**) resonance frequency of the TE_111_ mode. Modified after [27], with permission from Elsevier.

In order to monitor the stored amount of NH_3_ using microwaves, similar experimental procedures were performed in the test setup for powder measurements (Figure 5) as well as in the test bench for full-size automotive catalysts (Figure 8). Compared to the above-mentioned LF measurements, in these experiments initially all of the admixed NH_3_ is stored in the zeolite material. Hence, the stored amount can be calculated and be correlated with the microwave signal. The following tests were performed in the automotive catalyst setup on a serial type Cu-SSZ-13 zeolite coated on honeycomb-type cordierite substrate (Ford Motor Company) with 6.03 cm in diameter and 7.62 cm in length. In Figure 13, an experimental run at 250 °C with a total gas flow of 40 L/min and a background gas composition of 5% H_2_O and 7% O_2_ in N_2_ is shown. Figure 13a depicts the inlet (MFC data, dashed) and outlet (FTIR analysis data, solid) gas concentrations. From these data, the adsorbed amount of NH_3_ on the zeolite is calculated and is shown in gram per liter catalyst volume in Figure 13b. Figure 13c shows the corresponding resonance frequency of the TE_111_ mode, obtained from the reflection parameter *S*_11_. 500 ppm NH_3_ were admixed to the background gas at *t*_1_ until the catalyst became fully loaded (*t*_2_). At *t*_3_, the catalyst was purged in base gas and weakly bound NH_3_ was allowed to desorb slowly from the catalyst. Thus, the weakly bound and strongly bound NH_3_ species, which were observed in the IS-TPD (Figure 11), can be detected using microwaves. When NH_3_ was no longer detected downstream of the catalyst (*t*_4_), 500 ppm NO were added to fully convert the remaining NH_3_ on the catalyst according to the standard SCR reaction (Equation (1)). One may note the good correlation between the stored amount and the resonance frequency.

**Figure 14:**
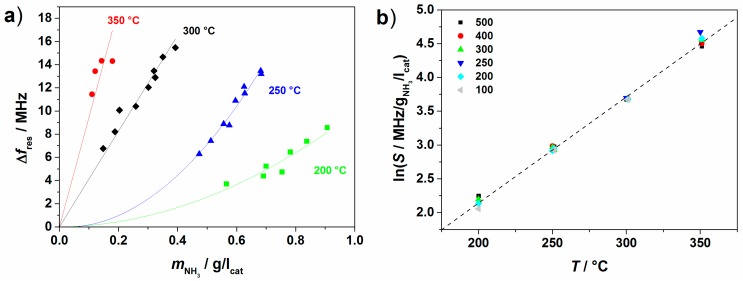
Cu-SSZ-13 honeycomb catalyst: (**a**) resonance frequency shift over stored amount of NH_3_ for different feed gas concentrations; (**b**) logarithm of the sensitivity of the high-frequency-based method over temperature for different feed gas concentrations of NH_3_ (in ppm). Adapted from [31], with permission from SAE.

Similar experiments were performed between 200 and 350 °C with NH_3_ feed concentrations between 25 and 500 ppm. In Figure 14a, the resonance frequency shift compared to the unloaded state (Δ*f*_res_ *= f − f*_0_) is plotted over the stored amount of NH_3_ on the Cu-SSZ-13 monolith catalyst at saturation (*t*_2_ in Figure 13). With reduced NH_3_ partial pressure and with increased temperature the adsorption capacity decreases. Furthermore, the microwave signal response increases with temperature, as the NH_3_-supported proton motion is thermally activated (see Figure 10). At higher temperatures, an almost linear correlation between the stored NH_3_ mass and the resonance frequency shift signal is observed, whereas at lower temperatures, a quadratic behavior adapts the data to a higher extent. By dividing the resonance frequency shift by the calculated stored amount, the sensitivity, *S* *=* Δ*f*_res_*/m*_NH3_, of the HF technique can be calculated. The logarithm of *S* over temperature is depicted in Figure 14b for all experiments. *S* increases linearly in this notation and does not range widely for the different feed gas concentrations.

**Figure 15:**
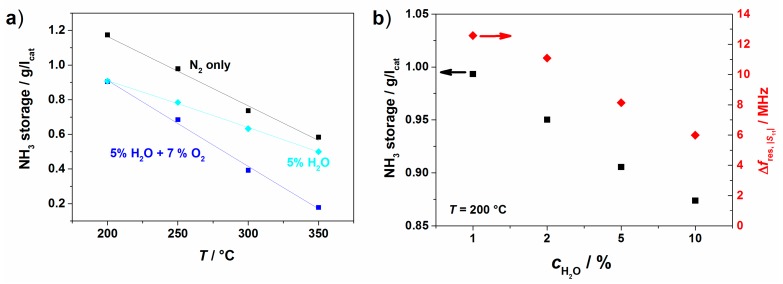
Cu-SSZ-13 honeycomb catalyst: (**a**) stored amount of NH_3_ over temperature for different background gas compositions; (**b**) stored amount of NH_3_ and resonance frequency shift for different H_2_O contents in background gas at 200 °C.

To evaluate the influence of the background gases (5% H_2_O and 7% O_2_ in the experiments shown in Figure 13 and Figure 14), similar tests were also conducted in N_2_ only and in a 5% H_2_O/N_2_ mixture. The stored amount of NH_3_ on the Cu-SSZ-13 monolith at an inlet concentration of 500 ppm is shown in Figure 15a over temperature for these gas compositions. Compared to the test with N_2_ only, the stored amount is reduced when 5% H_2_O is present, especially at lower temperatures. We assume that H_2_O molecules block the adsorption sites for NH_3_. When the gas contains H_2_O and O_2_, the adsorbed amount reduces further, in this case, especially at higher temperatures, due to the oxidation of NH_3_ with O_2_. These influences on the stored amount are also mirrored in the HF signal. In Figure 15b, the stored amount and Δ*f*_res_ are plotted for experiments with different H_2_O concentrations at 200 °C. As the stored amount decreases with increasing H_2_O concentration, Δ*f*_res_ is also reduced.

**Figure 16:**
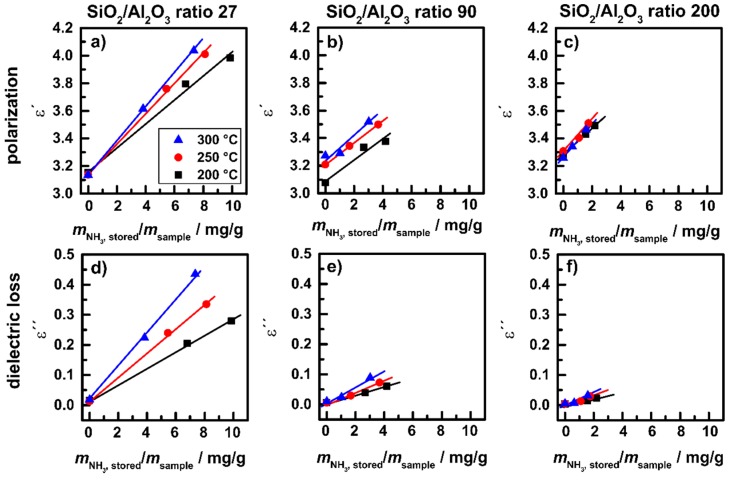
H-ZSM-5 powders: (**a**–**c**) polarization *ε*´; and (**d**–**f**) dielectric losses *ε*´´ over stored amount of NH_3_ for powders with different SiO_2_/Al_2_O_3_ ratios at 200, 250 and 300 °C. Reprinted from [30].

For a separated determination of both values of the complex permittivity of zeolite powders, experiments according to Figure 13 were performed on the powder resonator test bench (Figure 5) on H-ZSM-5 powders with SiO_2_/Al_2_O_3_ ratios of 27, 90, and 200. For these tests, a total gas flow of 500 mL/min was applied with 5% O_2_ in N_2_ serving as a background composition. 500 ppm NH_3_ were admixed for NH_3_ loading experiments, and for NH_3_ conversion tests, 175 ppm NO and 175 ppm NO_2_ were added to the background gas. In Figure 16a–c, the polarization *ε*′ and in Figure 16d–f, the dielectric losses *ε*" are plotted for the three samples over the relative stored ammonia mass (in relation to the sample mass). The values are shown for three steady state points of each measurement run, *i.e*., for the NH_3_-free sample at the beginning of the experiment (according to *t*_1_ in Figure 13), the total stored amount of NH_3_ at saturation (*t*_2_) and for the remaining strongly bound NH_3_ after free desorption (*t*_4_). In Figure 16a–c it becomes obvious that with growing stored mass of NH_3_, *ε*′ increases linearly and the slope is similar for all samples. The values of *ε*′ increase with temperature and decrease with Si/Al ratio. The dielectric losses *ε*" in Figure 16d–f show a linear behavior as well but reveal a higher dependency on temperature and on the SiO_2_/Al_2_O_3_ ratio. 

**Figure 17:**
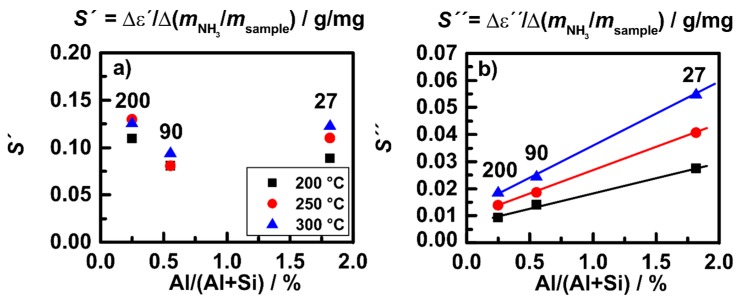
H-ZSM-5 powders: sensitivity of (**a**) *ε*′; and (**b**) *ε*" over Al content of the different samples at 200, 250 and 300 °C. Adapted from [30].

The sensitivity *S* (change in permittivity divided by the stored amount of NH_3_ as shown in Figure 16) is plotted in Figure 17 over the Al content of the samples. The sensitivity *S*′ (of *ε*′) seems to be unaffected by the SiO_2_/Al_2_O_3_ ratio, whereas the sensitivity *S*" (of *ε*") increases linearly with the Al content for each temperature and increases with increasing temperature. These data indicate that NH_3_ storage in the zeolite structure leads to structure-independent response of polarization effects related to the high polarity of the NH_3_ molecule. For the dielectric losses, which include conductivity mechanisms like proton hopping, the structure affects the response significantly. With increasing storage site distance (equivalent to a decrease in storage site number, *i.e*., with decreasing Al content) the sensitivity of the dielectric losses to the amount of NH_3_ decreases. This behavior fits to the observation in Figure 10 showing the transport of NH_4_^+^ in the observed temperature range between 200 and 300 °C. Extension of the studies on a broader temperature range is ongoing and is intended to monitor the catalytic activity of zeolite under technical operation conditions, *i.e*., up to 500 °C. Furthermore, the correlation with low-frequency impedance measurements is in progress, as will briefly be discussed in Section 4.

**Figure 18:**
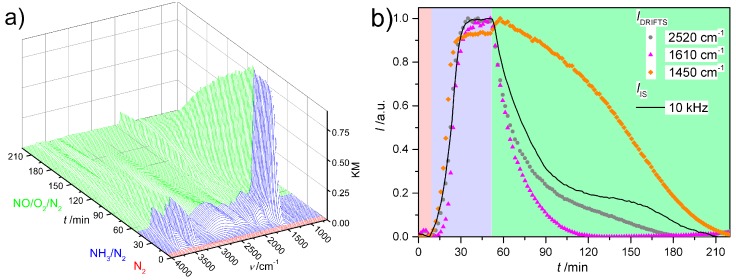
DRIFTS-IS study over a Fe-ZSM-5 SCR catalyst at 100 °C. (**a**) DRIFT spectra for Fe-ZSM-5 zeolite exposed to N_2_ (red), N_2_/NH_3_ mixture (blue) and NO/O_2_ mixture (green) in sequence; (**b**) comparison of normalized *I*_IS_ (green line) and *I*_DRIFTS_ (empty symbols) obtained under different gas conditions. The Fe-ZSM-5 catalyst was a commercial catalyst from Clariant, and with a SiO_2_/Al_2_O_3_ ratio of 30. Figures are adapted from [67], with permission from Elsevier.

Combining IS with DRIFTS is devoted to correlating the integral electric response of zeolite SCR catalyst with molecular processes on the surface under *in situ* conditions [67]. Figure 18a shows the DRIFT spectra collected over a commercial Fe-ZSM-5 catalyst after exposure to NH_3_ and NO/O_2_ mixture in sequence at 100 °C. After NH_3_ exposure, vibrational modes were clearly observed at 1610 cm^−1^ due to the stretching vibrations of NH_3_ bound either to Lewis sites or to extra-framework Al, and at 1450 cm^−1^ due to the deformation vibrations of NH_4_^+^ resulting from NH_3_ directly bound to Brønsted sites [67]. The weak bands at 2520 cm^−1^ are attributed to the vibrations of NH_3_ weakly bound to those strongly bound NH_3_ on Brønsted sites by hydrogen bonds, *i.e*., the so-called secondary coordinated NH_3_ [67]. Exposure of the NH_3_-saturated Fe-ZSM-5 to NO/O_2_ mixture led to the decrease of these NH_3_-related bands, and the appearance of new band at 1875 cm^−1^ (not shown) due to the vibration of NO bounded to Fe sites.

From the comparison of the normalized *I_IS_* and *I_DRIFTS_* (Figure 18b), one can see that the build-up of NH_3_ on Fe-ZSM-5 led to a rapid increase of *I_IS_* indicating significantly enhanced conductance as reported in previous studies [19,21,27]. Both, *I_IS_* and *I_DRIFTS_* reached maximal values within 10 min upon exposure to NH_3_, and remained in a stable state, indicating that the zeolite catalyst was fully saturated by NH_3_. *I_IS_* decreased immediately upon switching the gas mixture from NH_3_ to NO/O_2_ due to the reduced amounts of NH_3_ species. This was confirmed by the decreased *I_DRIFTS_* values. Both, *I_IS_* and *I_DRIFTS_* at 2520 cm^−1^ decayed asymptotically in the first 40 min and then sigmoidally to values of the NH_3_-free state, demonstrating the decisive impact of the secondary NH_3_ species on the conductance of Fe-ZSM-5 zeolite catalyst. Afterwards, the impedance behavior was dominated by strongly bound NH_3_ in the absence of secondary NH_3_ species [71].

## 4. Future Directions

The examples given in this work illustrate that the three methods applied to analyze the gas sensing and catalytic properties of zeolites address different length scales and frequency ranges. While LF IS probes the translational motion of charge carriers (in this particular case of protons) up to the length scale of multiple zeolite grains or crystals in sub-millimeter-thick films, HF IS analyzes local charge transport properties based on ion displacement current as well as electronic and dipolar polarization. The present state of knowledge implies that the NH_3_-sensing effect in both frequency ranges is caused by the acid-base interaction between NH_3_ and Brønsted acidic protons bound to the polyanionic zeolite lattice. The long range (monitored at low frequencies) motion of protons is thus characterized by thermally activated hopping along a characteristic energy landscape, which is affected by the uptake of the guest molecules through the formation of solvent complexes. HF IS addresses the local motion of charge carriers, *i.e*., the on-site motion of protons between oxygen atoms coordinated to the Al site in the zeolite lattice, and the polarization at smaller length scales, including molecular (dipolar) and electronic polarization. The main challenge in terms of uniting the results derived from the two frequency ranges is to develop a physicochemical model which takes into account characteristic molecular signatures as well as microstructural effects. 

The characteristic molecular signature can be partially obtained from DRIFTS analyses, which probe the molecular length scale. The correlation of IS and DRITFS, as discussed here, is the first step toward bridging the scales between processes appearing in micron-sized (macroscopic) samples and at the true molecular level. However, so far, a rather unexplored aspect is the effect of the sample microstructure in low-frequency and high-frequency measurements. It might be assumed that grain boundaries or other extended structural defects, which are unavoidably present in macroscopic samples, have an influence on translation proton motion as well as on the overall polarization. This is a drawback of any kind of volume-integrating analyses. Therefore, it would be highly desirable to study the electrical properties of individual zeolite crystals, which would eventually allow the discrimination of intrinsic and interfacial effects. 

In principle, local probe techniques are available to electrically address nanoscale objects, by means of either a conductive atomic force microscopy [72] or lithographic techniques, such as electron-beam-induced deposition to form nano-sized electrodes [73]. Among these, a very promising approach is the use of nanorobotics systems installed in a scanning electron microscope (SEM), which have previously been used to study the electrical properties of inorganic microcrystals [74] or nanowires [75]. Details of the set-up are described elsewhere [76].

**Figure 19:**
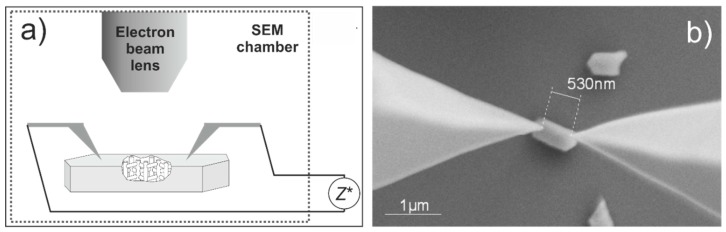
(**a**) Measuring configuration for electric measurement on single zeolite crystal; (**b**) H-ZSM-5 crystal electrically contacted by two metallic tips. The spacing between the tips in (**b**) is about 530 nm.

By means of this setup, first, orientating 2-point and 4-point measurements on individual zeolite crystals has been performed. Figure 19 shows an SEM image of a single H-ZSM-5 crystal, which is electrically contacted by two metallic tips. In such a measuring configuration, conductance data could be obtained, e.g., from a Cu-exchanged ZSM-5, on which currents in the nA range at 1 V could be measured, which is a conductance range that is very accessible with this setup [77]. These preliminary measurements are the starting point of a systematic study on different zeolite materials, which is promising in terms of contributing to a refined understanding of how microstructural effects may contribute to the overall electrical response.

In the case of LF IS measurements, several parameters of the zeolite layer on IDE structures, including the thickness, packing density, or homogeneity, influence the sensing response to different extents [71]. Close contact (less empty space) between the zeolite grains as well as the zeolite layer and IDE was found to be beneficial and crucial in gas sensing. By applying advanced methods such as screen printing and aerosol deposition [78], highly reproducible IDE sensor chips can be prepared without significant differences with respect to the above-mentioned parameters. According to comparative measurements using different IDE sensor chips (with the same zeolite material) prepared in the same batch or in different batches, the screen-printed, zeolite-based sensors result in reproducible and reliable sensing response. Nevertheless, sensing performance may be further improved by optimizing the preparation of zeolite films.

## 5. Conclusions

In zeolites, when they are applied as gas-sensing materials, interaction of the gas molecules with mobile cations, which are non-covalently bound to the zeolite lattice, can be monitored at different length scales (from the true molecular scale to centimeters) and frequency scales (from IR down to a low frequency approaching the quasi-DC limit). In H-form zeolites, low-frequency (mHz to MHz) and high-frequency (GHz) impedance measurements probe the translational and local mobility of protons, respectively, while DRIFTS detects characteristic vibrational modes of the guest molecules closely interacting with zeolite. The combination of these methods is applied to monitor the DeNO_x_-SCR processes *in situ*, which correlates the gas-sensing and the catalytic properties of H-form and metal-promoted zeolites. 

This review thereby has summarized how the different experimental methods can be combined to develop a refined physicochemical model for the sensing mechanism as well as for the elementary catalytic processes occurring during DeNO_x_-SCR. The electrical properties of zeolites were changed upon interacting with guest molecules, in particular with NH_3_, which can be analyzed by IS in a broad frequency range. While IS in a low frequency range can be applied to analyze the translational motion of protons and the related characteristic energy landscape, IS in a high frequency range allows the discriminating of on-site motion of protons and the local polarization. These fundamental understandings of proton motion lead to the successful application of zeolites as impedimetric NH_3_ sensor and, more interestingly, allow determining the NH_3_ storage in zeolite catalyst from micrometer-thick film to technically relevant size. A combination of low-frequency IS with DRIFTS revealed that both strongly and weakly bound NH_3_ species contribute to the overall proton conductivity of zeolite catalyst, thus correlating the sensing properties with elementary catalytic processes and, furthermore, enabling the *in situ* monitoring of DeNO_x_-SCR reaction on zeolite catalyst. Preliminary local probe measurements were performed over single zeolite crystals in nanometer size, which is expected to address the microstructural effects on the proton motion in zeolites.

Although this correlation is far from sufficient to develop a unified model suited to bridge the different time and length scales, we hope that the results presented in this review will stimulate a discussion among scientists working in the fields of gas sensing, heterogeneous catalysis and molecular spectroscopy in order to gain a refined understanding of the interplay of sensing and catalytic properties in general.
